# An epigenetic mechanism for over-consolidation of fear memories

**DOI:** 10.1038/s41380-022-01758-6

**Published:** 2022-09-21

**Authors:** Riccardo Barchiesi, Kanat Chanthongdee, Michele Petrella, Li Xu, Simon Söderholm, Esi Domi, Gaelle Augier, Andrea Coppola, Joost Wiskerke, Ilona Szczot, Ana Domi, Louise Adermark, Eric Augier, Claudio Cantù, Markus Heilig, Estelle Barbier

**Affiliations:** 1 Center for Social and Affective Neuroscience, Department of Biomedical and Clinical Sciences, Linköping University, S-581 85 Linköping, Sweden; 2 Department of Physiology, Faculty of Medicine Siriraj Hospital, Mahidol University, Bangkok, Thailand; 3 Wallenberg Centre for Molecular Medicine, Linköping University, Linköping, Sweden; 4 Department of Biomedical and Clinical Sciences, Division of Molecular Medicine and Virology; Faculty of Medicine and Health Sciences, Linköping University, Linköping, Sweden; 5 Addiction Biology Unit, Department of Psychiatry and Neurochemistry, Institute of Neuroscience and Physiology, The Sahlgrenska Academy, University of Gothenburg, Gothenburg, Sweden

**Keywords:** Neuroscience, Molecular biology

## Abstract

Excessive fear is a hallmark of anxiety disorders, a major cause of disease burden worldwide. Substantial evidence supports a role of prefrontal cortex-amygdala circuits in the regulation of fear and anxiety, but the molecular mechanisms that regulate their activity remain poorly understood. Here, we show that downregulation of the histone methyltransferase PRDM2 in the dorsomedial prefrontal cortex enhances fear expression by modulating fear memory consolidation. We further show that *Prdm2* knock-down (KD) in neurons that project from the dorsomedial prefrontal cortex to the basolateral amygdala (dmPFC-BLA) promotes increased fear expression. *Prdm2* KD in the dmPFC-BLA circuit also resulted in increased expression of genes involved in synaptogenesis, suggesting that *Prdm2* KD modulates consolidation of conditioned fear by modifying synaptic strength at dmPFC-BLA projection targets. Consistent with an enhanced synaptic efficacy, we found that dmPFC *Prdm2* KD increased glutamatergic release probability in the BLA and increased the activity of BLA neurons in response to fear-associated cues. Together, our findings provide a new molecular mechanism for excessive fear responses, wherein PRDM2 modulates the dmPFC -BLA circuit through specific transcriptomic changes.

## Introduction

Normal fear elicits adaptive responses aimed at escaping life-threatening events [1, 2]. In contrast, excessive fear responses become maladaptive and are characteristic of fear-related disorders such as post-traumatic stress disorder and several anxiety disorders [3]. Pathology of memory processes involved in learned fear can lead to amplified behavioral responses that fail to extinguish despite the absence of a relevant threat [4]. Identifying the neural circuits and molecular mechanisms underlying excessive fear memory may identify molecular pathways that can be targeted by mechanistic treatments.

Research using fear conditioning has identified brain structures involved in fear memory processing [5, 6]. Among these, the amygdala complex, including the central amygdala (CeA) and basolateral amygdala (BLA), is critical for Pavlovian fear conditioning [7]. The CeA is involved in the expression of conditioned fear responses, whereas the BLA acts as a primary site where associations between conditioned and unconditioned stimuli are formed and stored [8]. The amygdala is extensively interconnected with the prefrontal cortex (PFC), a brain region important for emotion regulation [9]. The PFC, including dorsomedial prefrontal cortex (dmPFC; prelimbic and cingulate cortex) and ventromedial prefrontal cortex (infralimbic), is also thought to participate in fear memory processing [10, 11]. In particular, activation of projections from the dmPFC to the BLA has been associated with top-down regulation of fear expression [12–15]. However, the molecular mechanisms that regulate the dmPFC -BLA pathway modulation of fear memory processing have yet to be fully understood.

Growing evidence points toward a role of epigenetic mechanisms in regulating fear memory processes [16, 17]. Past experiences including stressful events can lead to a “reprogramming process” through gene expression changes. Epigenetic regulation is thought to be a key mechanism that alters transcription and translation [18] in an experience-dependent manner [19–21]. Broad dysregulations of gene expression mediated by epigenetic processes may therefore link traumatic stress exposure to the development of stress-related disorders.

We previously found that downregulation of the histone methyltransferase PR containing domain 2 (PRDM2), by a history of alcohol dependence, was associated with increased stress responses. PRDM2 promotes gene silencing through the addition of a methyl group at histone H3 lysine 9 [22]. PRDM2 is strongly enriched in the brain and is selectively expressed in neurons of the dmPFC, which suggests a role of this epigenetic enzyme in neuronal function [23]. Accordingly, we found that *Prdm2* knock-down (KD) in the dmPFC potentiated stress-induced relapse to alcohol seeking [23]. A growing literature indicates a link between excessive alcohol use and fear-related disorders at both behavioral and neural levels [24–28]. Here, we, therefore, hypothesized that *Prdm2* deficiency in the dmPFC may contribute to the development of pathological fear by promoting gene expression changes in fear-related brain circuits.

To test this hypothesis, we knocked down *Prdm2* in the rat dmPFC, and assessed the effects on acquisition, expression, and extinction of conditioned fear. Given the critical role of dmPFC projections to the BLA in cued conditioned fear [12, 13, 29, 30], we next used a projection-specific strategy to determine whether *Prdm2* KD regulates fear through this neuronal pathway. We then used viral translating ribosomal affinity purification (vTRAP) with high throughput RNA sequencing (RNAseq) to identify the downstream molecular consequences of *Prdm2* KD in a circuit-specific manner. Because this analysis identified a broad upregulation of transcripts encoding proteins involved in regulation of synaptic activity, we finally investigated the effects of *Prdm2* KD on glutamatergic inputs to the BLA using patch-clamp recordings in amygdala slices and measured the activity of dmPFC-BLA neurons during fear memory testing with in vivo fiber photometry.

## Materials and methods

### Animals

Adult male Wistar rats (200–225 g, Charles River, Germany) were housed under a reverse light cycle with unlimited food and water. Procedures were in accordance with the National Committee for animal research in Sweden and approved by the Local Ethics Committee for Animal Care and Use at Linköping University.

### Behavioral testing

Nine batches of rats were used in this study (*N* = 268). Animal grouping was assigned randomly. In experiment 1 (*n* = 17 scrambled and 20 *Prdm2* KD), rats were tested for acquisition, expression (after 24 h), and extinction of fear memory. In experiment 2 (scrambled: *n* = 12 and *Prdm2* KD *n* = 12), rats were tested for expression of fear memory 1 week after conditioning as well as for context generalization and foot shock sensitivity. In experiment 3 (*n* = 17 scrambled and 20 *Prdm2* KD), we replicated the effect of *Prdm2* KD increased fear expression 24 h following cued fear conditioning. Prior to undergoing fear conditioning, rats were tested for anxiety in the EPM and locomotor activity. Plasma corticosterone levels were measured at baseline, after conditioning, and after testing the expression of fear memory. One week after the fear expression test, rats were euthanized, and the dmPFC was collected for gene expression analysis. In experiment 4 (scrambled: *n* = 20 and *Prdm2* KD *n* = 18), rats were conditioned to the fear stimulus 1 week prior to the viral-mediated KD of *Prdm2* and tested for fear expression one month after the surgery. In experiment 5 (scrambled: *n* = 12 and *Prdm2* KD *n* = 12), rats were tested for novel object recognition. In experiment 6 (scrambled: *n* = 20 and *Prdm2* KD *n* = 19), the effects of *Prdm2* KD in neurons specifically projecting to the BLA were investigated on the expression of fear memory 24 h after conditioning. In experiment 7 (scrambled: *n* = 18 rats; pools of 3 dmPFC and *Prdm2* KD *n* = 18 rats; pools of 3 dmPFC), we used vTRAP to analyze gene expression following *Prdm2* KD specifically in the neurons projecting from the dmPFC to the BLA. In experiment 8 (scrambled: *n* = 14 cells from 5 rats and *Prdm2* KD *n* = 15 cells from 6 rats) we used ex vivo electrophysiology to investigate changes in glutamate release to BLA neurons following *Prdm2* KD. Finally, in experiment 9 (scrambled: *n* = 9 and *Prdm2* KD *n* = 13) we used in vivo fiber photometry to further investigate the consequences of *Prdm2* KD in the BLA. An overview of the experiments conducted in this study is given in Supplementary Fig. S1.

#### Cued fear conditioning

During fear conditioning, rats were conditioned in a chamber with specific visual and odor cues and exposed to six trials of 2 s, 1 mA foot shocks associated with a 30 s neutral cue tone (2.9 kHz, 80 dB; inter-trial interval: 3 min; Med Associates Inc., St Albans, VT, USA). Rats were tested for the expression of fear memory in a chamber with different visual and odor cues and exposed to 6 × 30 s cue tones (see supplemental methods for details). Extinction was investigated by repeating the fear expression test over two more days. Fear expression was measured as % time spent freezing during the 30 s tone by two trained experimenters unaware of the rat’s group identity at the time of the scoring. Expression and extinction of fear memory are presented as an average of block 1 (tones 1 and 2) during the test sessions each day, as extinction may be observed within session.

#### Novel object recognition

Objects were custom-built and made interactive (climbable) to increase exploration time and memory acquisition, as novel object recognition is normally used to study short-term memory [31] (Supplementary Fig. S3). Rats were allowed 10 min to familiarize themselves with two copies of either object A or object B. On the following day, they were tested for novel object recognition for 5 min by replacing one of the familiar objects with one that was novel. Data are presented as a recognition index, defined as: time spent exploring novel object/(time spent exploring novel + familiar object).

#### Elevated plus maze

Basal anxiety-like behavior was measured using the elevated plus maze paradigm (EPM) as previously described [32, 33]. Data are represented as % time in open arm, i.e., time in open arm/(time in open arm + time spent in the closed arm) *100.

#### Locomotor activity

Locomotor activity was tested for 30 min under ambient light levels (190–210 lux) in sound attenuated chambers (43 × 43 cm) equipped with an infrared beam detection system (Med Associates Inc.). Data are presented as cumulative distance traveled in 5 min intervals.

#### Foot shock sensitivity

Foot shock sensitivity thresholds were tested in Med Associates boxes. Rats were exposed to 0.5 s foot shocks in 0.1 mA increments, starting from 0.1 mA, and the retraction of 1, 2, and 4 paws was scored by a blinded observer.

### Surgeries

#### *Prdm2* dmPFC KD

Rats received bilateral infusions into the dmPFC (0.25 µl/infusion; rate: 0.1 µl/min; anteroposterior: +3 mm, mediolateral: ±0.6 mm, dorsoventral: −3.5 mm [34] of an adeno-associated virus (AAV) containing a short hairpin RNA targeting *Prdm2* (AAV9.HI.shR.ratPrdm2.CMV.ZsGreen.SV40; GGAGCCCAAGTCCTTGTAAAT; titer: 5.6E13 GC/mL; UPenn Core Facility, Philadelphia, PA) or a scrambled control (AAV9.HI.shR.luc.CMV.ZsGreen.SV40; titer: 9.2E13 GC/mL; UPenn Core Facility, PA).

#### *Prdm2* KD dmPFC-BLA

Rats received bilateral infusions into the dmPFC (0.25 µl/injection; rate: 0.1 µl/min; coordinates: anteroposterior: +3 mm, mediolateral: ±0.6 mm, dorsoventral: −3.5 mm [34] of an AAV containing a Cre-dependent microRNA targeting *Prdm2* (AAV9.hSyn.DIO.-mcherry.miR.Prdm2.WPRE; GGAGCCCAAGTCCTTGTAAAT; titer: 3.24E13 GC/ml; UPenn Core Facility, PA) or a scrambled control (AAV9.HI.shR.luc.CMV.ZsGreen.SV40). Rats also received bilateral infusions of a retrogradely transported AAV encoding Cre (0.5 µl/infusion; rate: 0.1 µl/min; AAV-retro2-hSyn1-EGFP_iCre-WPRE-hGHp(A); titer: 7.8 × 10E12 GC/mL; Addgene, Watertown, MA) into the BLA (anteroposterior: −2.4 mm, mediolateral: ±5 mm, dorsoventral: −8.4 mm [34]).

#### Fiber photometry BLA

Rats received bilateral infusions into the dmPFC of an AAV-vector containing an shRNA targeting *Prdm2* or a scrambled control as described above. Rats also received unilateral infusion of an AAV encoding GcaMP6s (0.5 µl/infusion; rate: 0.1 µl/min; AAV9.CaMKII.GcaMP6s.WPRE.SV40; titer: 2.1E13 GC/mL; Addgene, Watertown, MA) into the BLA (anteroposterior: −2.4 mm, mediolateral: ±5 mm, dorsoventral: −8.4 mm). Following vector infusions, a 400 µm core, 0.66NA borosilicate optical fiber attached to a titanium M3 receptacle (Doric Lenses, Quebec City, Canada) was implanted dorsal to the BLA (anteroposterior: −2.4 mm, mediolateral: ±5 mm, dorsoventral: −8.5 mm). The receptacle was secured to the skull with a combination of skull screws, superglue, and black Ortho-Jet dental acrylic (Riss-Dental, Hanau, Germany).

### Fiber photometry calcium imaging

Rats were habituated to being connected to a fiber patch cord for several days prior to the fear conditioning session. Cued expression of fear memory was again assessed 24 h after conditioning, and GCaMP6s-emitted fluorescence as a proxy for calcium activity was measured during the entire session using previously described methods [35–37], with minor modifications. In brief, GCaMP6s were excited at two wavelengths (465 nm, calcium-dependent signal, and 405 nm isosbestic control) by light originating from two sinusoidally modulated LEDs (330 Hz and 210 Hz for 465 and 405 nm, respectively), reflected off dichroic mirrors (4-ports fluorescence minicube; Doric Lenses), and coupled into a 400 μm 0.57NA optical fiber patch cord that in turn was connected to the fiber implant. Light intensity for both wavelengths was adjusted to 10–15 µW at the tip of the patch cord. Emitted signals from both channels then returned through the same optical fiber and were acquired at 6.1 kHz using a photosensor build into the fluorescence minicube, demodulated (lock-in amplification) and digitized at 1017.3 kHz, and recorded by a real-time signal processor (RZ5D; Tucker Davis Technologies, Alachua, FL, USA). Behavioral timestamps of tone (CS+) onset and offset were digitized by TTL input to the real-time signal processor from the Med-Associates behavioral chambers.

For further offline analysis, only rats with correct fiber placement and GcaMP expression directly underneath the fiber tip (post-mortem inspection) as well as observable calcium activity (visual inspection of whole session traces) were included. This analysis was performed using a custom-written Graphical User Interface (GUI) based on Python scripts. The raw 465 nm and 405 nm signals were first down-sampled (50×) and smoothed (zero-phase moving average filter with window size 10 samples). Next, peri-event histograms were created trial-by-trial with a time window encompassing −30 s and 40 s surrounding tone onset. Finally, data were detrended to remove movement, photo-bleaching, and fiber bending artifacts. Per trial, signals from both channels were independently fitted to a time curve using linear polynomial regression to generate a predicted signal for both channels. Subtracting the predicted signal from the measured raw signal resulted in a ∆*F* for each channel, which subsequently was normalized through division by the channel’s predicted signal, resulting in ∆*F*/*F*. Detrended signals from the 465 nm and 405 nm channels were then subtracted from one another to calculate a normalized calcium-dependent GCaMP fluorescent signal (% ∆*F*/*F*).

### vTRAP and RNA sequencing

To identify the molecular mechanisms downstream of *Prdm2* KD, specifically in neurons projecting from the dmPFC to the BLA, we used the viral translating ribosomal purification vTRAP method. In this method, a construct encoding an enhanced green fluorescent protein (EGFP)-tagged ribosomal subunit (L10a) is selectively expressed in neuronal populations of interest, using e.g., the Cre/lox system. The EGFP-tagged subunit is then incorporated into the ribosomes of transfected cells. These tagged ribosomes, together with the translating RNA bound to them, can then be isolated with immunoprecipitation and subjected to gene expression analysis using RNAseq. The main advantage of using TRAP is that the mRNA associated with the ribosomes is in the process of translation. Translation occurs after many of the gene expression regulatory events have already taken place, and translating mRNA will therefore more closely correlate with the protein levels [38].

Rats received bilateral infusions in the dmPFC of a viral cocktail with 1:4 parts of an AAV9 containing an shRNA targeting *Prdm2*, or a scrambled control, and 3:4 parts of an AAV5 encoding a Cre-dependent, EGFP-tagged ribosomal subunit (EGFP-L10a; AAV5-FLEX-EGFPL10a; titer: 7 × 10¹² vg/mL). Rats also received bilateral infusions of the AAV2-retro encoding Cre (0.5 µl/infusion; AAV-retro/2-hSyn1-mCherry_iCre-WPRE-hGHp(A); titer: 5.0 × 10E12 vg/mL) into the BLA (anteroposterior: −2.4 mm, mediolateral: ±5 mm, dorsoventral: −8.4 mm) [34]. Isolation of the dmPFC-BLA projecting neurons was performed as previously described [39]. In brief, dmPFC was dissected, and samples were homogenized in lysis buffer (10 mM HEPES-KOH; pH 7.4, 150 mM KCl, 5 mM MgCl_2_, 0.5 mM DTT, 100 mg/mL cycloheximide, RNasin; Promega and SUPERase-In™, Thermo Fisher Scientific, Waltham, MA, USA; RNase inhibitors, and Complete-EDTA-free protease inhibitors, Roche, Basel, Switzerland). Samples underwent three centrifugation steps in which 10% NP-40 and 300 mM DHPC were added to the supernatant after 1st and 2nd centrifugation, respectively. Polysome immunoprecipitation was performed using monoclonal anti-EGFP antibodies (Memorial Sloan-Kettering Monoclonal Antibody Facility; clone names: Htz-GFP-19F7 and Htz-GFP-19C8) bound to biotinylated-Protein L (Pierce; Thermo Fisher Scientific)-coated streptavidin-conjugated magnetic beads (Life Technologies). RNA was then purified using the Absolutely RNA Nanoprep kit (Agilent Technologies, Santa Clara, CA, USA). RNA concentration and integrity were measured using the bioanalyzer RNA 6000 Pico assay (RNA concentration: ∼990 pg/µl, RIN 9.5–10) and sent to the National Genomics Infrastructure (NGI, Sweden) for RNAseq.

#### Sequencing data analysis

Samples were sequenced on NovaSeq6000 (NovaSeq Control Software 1.7.0/RTA v3.4.4; Illumina, Inc., San Diego, CA, USA) with a 151nt(Read1)-10nt(Index1)-10nt(Index2)-151nt(Read2) setup using ‘NovaSeqXp’ workflow in “S4” mode flowcell. The Bcl to FastQ conversion was performed using bcl2fastq_v2.20.0.422 from the CASAVA software suite. The quality scale used is Illumina 1.8.

Sequencing data quality was assessed using FastQC and Preseq. Quality trimming and trimming to remove adapter sequences was done with TrimGalore! and Cutadapt in paired-end trimming mode and with quality Phred score cutoff 20. The STAR aligner software was used to align the trimmed sequencing reads to Ensembl reference rat genome Rnor_6.0 with associated annotation data. Alignment quality was assessed using Qualimap and mapping of aligned reads to genomic features was performed with featureCounts. Results were summarized using MultiQC.

Downstream analyses were done with the R programming language and Rstudio. Variance and library size normalization of the count data was done by applying a variance stabilizing transformation (VST) before principal component analysis. Differential gene expression analysis was performed with R package DESeq2. A false discovery rate adjusted *p* value below 0.05 was used as cutoff for statistical significance.

We used the Ingenuity Pathway Analysis (IPA; Agilent Technologies) software to identify interconnected genes in a pathway. The database that supports IPA contains over 7 million findings and is continually updated. The algorithm used by IPA allows us to identify genes enriched in a given pathway. It also provides pathway activation/inhibition predictions, therefore indicating whether the identified gene expression changes can lead to activation or inhibition of a specific pathway. To further explore the underlying patterns of our RNA-seq data, we performed a weighted gene co-expression network analysis (WGCNA), using the WGCNA R package with default parameters. Through this approach, a weighted gene co-expression network was constructed based on VST-normalized read count data. Modules of interconnected, highly co-expressed genes were identified using a minimum module size of 30 genes, and modules with similar expression profiles (correlation > 0.75) were merged. Differential expression analysis was performed by fitting a linear model to the data using the limma package in R, comparing the expression profiles of each module between Prdm2 KD and scrambled control samples. Module genes were analyzed for functional enrichment based on data from the Gene Ontology knowledgebase, using the R package clusterProfiler.

### RNAscope fluorescent in situ hybridization

After completion of experiments 3 and 6, brains were removed and flash frozen. 12 µm brain sections were collected at the dmPFC level and kept at −80 °C until use. In situ hybridization was performed following the RNAscope Fluorescent Multiplex Kit User Manual (Advanced Cell Diagnostics, Newark, CA) and as previously described [23]. The *Prdm2* probe (accession number NM_001077648.1) was purchased from Advanced Cell Diagnostics (Newark, CA, USA). Briefly, sections were incubated at 40 °C with a series of 4 probes designed to amplify transcripts to a point where they can be individually quantified. This includes the *Prdm2* target probe, a preamplifier probe, an amplifier probe, and the fluorescently labeled probe Atto 550 (red) in the C1 channel to visualize *Prdm2* transcripts. Microphotographs for quantification were obtained using a confocal microscope at 20× magnification (Zeiss LSM 700; Carl Zeiss AG, Jena, Germany). *Prdm2* mRNA levels were assessed as total pixels of the fluorescent signal. We assumed that each pixel represents a single molecule of mRNA. Total *Prdm2*-positive pixels were measured after automatically adjusting the threshold using ImageJ software (National Institutes of Health, Bethesda, MD, USA) [40].

### Slice preparation and ex vivo electrophysiology

Brains were quickly removed and placed into an ice-cold N-methyl-D-glucamine (NMDG)-based cutting solution containing (in mM): 92 NMDG, 20 HEPES, 25 glucose, 30 NaHCO_3_, 1.2 NaH_2_PO_4_, 2.5 KCl, 5 sodium ascorbate, 3 sodium pyruvate, 2 thiourea, 10 MgSO_4_, and 0.5 CaCl_2_ (310 mOsm, pH 7.4). Acute coronal brain slices (250 μm thick) containing either the PFC or the BLA were obtained using a vibratome (Leica VT1200 S, Leica Biosystems Inc., IL, USA). After cutting, slices were transferred to a holding chamber filled with a pre-warmed (∼34 °C) holding solution containing (in mM): 92 NaCl, 20 HEPES, 25 glucose, 30 NaHCO_3_, 1.2 NaH_2_PO_4_, 2.5 KCl, 5 sodium ascorbate, 3 sodium pyruvate, 2 thiourea, 1 MgSO_4_, and 2 CaCl_2_ (310 mOsm, pH 7.4). Subsequently, the holding solution was maintained at room temperature and after >1 h of recovery, a single slice was transferred to the recording chamber and continuously perfused at a flow rate of ∼2.0 ml/min with warmed (∼30–32 °C) artificial cerebrospinal fluid (aCSF, in mM): 125 NaCl, 2.5 KCl, 1.25 NaH_2_PO_4_, 1 MgCl_2_, 11 glucose, 26 NaHCO_3_, 2.4 CaCl_2_ (310 mOsm, pH 7.4). All solutions were saturated with 95% O_2_ and 5% CO_2_. Spontaneous EPSCs (sEPSCs) were recorded using borosilicate glass patch pipettes (2.5–3.0 MΩ; Harvard Apparatus, MA, USA) containing (in mM): 135 K-gluconate, 20 KCl, 10 HEPES, 0.1 EGTA, 2 MgCl_2_, 2 Mg-ATP, 0.3 Na-GTP (290 mOsm, pH adjusted to 7.3 using KOH). Evoked EPSCs (eEPSCs) were recorded using a Cs-based intracellular solution containing (in mM): 140 Cs-methanesulfonate, 5 NaCl, 1 MgCl, 10 HEPES, 0.2 EGTA, 2 Mg-ATP, 0.5 Na-GTP, 5 QX-314 chloride (290 mOsm, pH adjusted to 7.3 using CsOH). Paired-pulse ratio was analyzed as the ratio between eEPSC2 and eEPSC1 (0.05 Hz, 50 ms interpulse interval, 50 μs stimulus duration). The inverse square of the coefficient of variation (1/*CV*^2^) was measured as the ratio between the square of mean amplitude and the variance (*μ*^2^/*σ*^2^) of 20 consecutive eEPSCs (0.05 Hz). The variance-to-mean ratio (VMR) was measured as the variance divided by the mean amplitude (*σ*^2^/*μ*) of 20 consecutive eEPSCs (0.05 Hz). AMPA/NMDA ratio was measured by dividing the peak amplitude of the AMPAR-mediated current (measured at −70 mV) with the NMDAR-dependent component (measured at +40 mV, 50 ms after the onset of the eEPSC) in the absence of glutamatergic receptor blockers. All recordings were carried out in voltage-clamp mode with a holding potential of −70 mV and in presence of the GABARs blockers picrotoxin (100 μM) and CGP55845 (2 μM). The estimated junction potential was 11 mV for K-Gluc and 8.5 mV for Cs-based intracellular solution and was not compensated during electrophysiological recordings. Results were analyzed using Mann-Whitney tests, and all reagents and drugs were obtained from Thermo Fisher Scientific.

### Statistical analysis

Behavioral and fiber photometry data were analyzed using Statistica 13.0 (TIBCO Software, Palo Alto, CA, USA) and graphs produced using Prism 9.1.2 (Graphpad Software LLC., San Diego, CA, USA). Normality was checked using Shapiro-Wilk testing. Peak calcium response data had to be log-transformed to conform to normal distribution before analysis. Homogeneity of variance was assessed using Levene’s test. Data did not violate the assumption of homogeneity and were therefore analyzed using unpaired student *t*-test, one-way ANOVA, or two-way repeated measures ANOVA, as appropriate. The accepted level of significance for all tests was *p* < 0.05. Data are presented as means ± SEM, unless otherwise stated. Analysis of RNA sequencing data and electrophysiological recording are described above. Sample size for each experiment was based on the variation observed in prior, similar experiments and pilot studies. Rats with viral injection/optical fiber that was outside of the targeted area were removed from the study.

## Results

### *Prdm2* KD in the dmPFC induces a long-lasting increase in the expression of cued conditioned fear

**Fig. 1:**
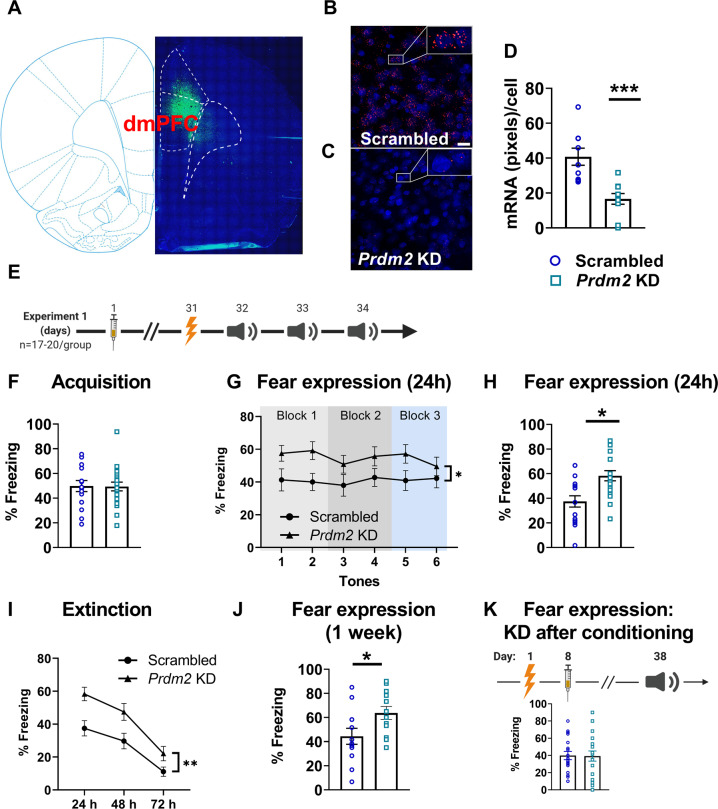
Knock-down (KD) of *Prdm2* in the dorsomedial prefrontal cortex (dmPFC) causes a lasting increase in fear expression. **A** Representative tile scan of virus injection site and spread. **B**, **C** Representative images used for RNAscope quantification. **D** KD of *Prdm2* induces a significant downregulation of *Prdm2* in the dmPFC. **E** Experimental timeline: On day 1, rats received bilateral infusion of an AAV9 containing either a shRNA-*Prdm2* or a scrambled control. Rats were then tested for fear acquisition (day 31), fear expression (day 32), and fear extinction (day 33–34). **F** KD of *Prdm2* did not affect the acquisition of fear memory (acquisition of fear memory is presented as an average of tones 2–6 during the conditioning session), but **G** significantly increased fear expression 24 h after conditioning (indicated by the % freezing ±SEM; *N* = 17–20/ group). **H** Average of the first 2 tones from the fear expression test. **I** *Prdm2* KD did not affect the rate of extinction, indicated by the interaction for time x group not being significant (i.e., similar slopes). **J** In a separate batch (*N* = 12/group), *Prdm2* KD was found to increase fear expression also 1w after conditioning. **K** When *Prdm2* was knocked down 1 week after the acquisition of fear memory (*N* = 18–20/group), no effect was observed on fear expression measured 1 month later. *p < 0.05; **p < 0.01; *p* < 0.001.

**Fig. 2:**
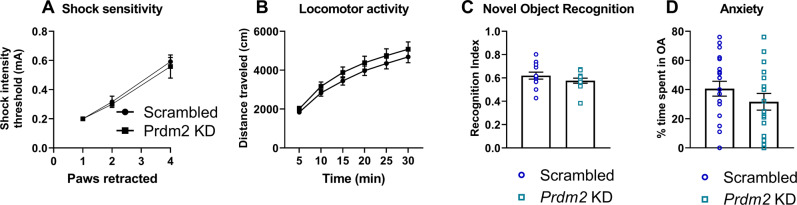
*Prdm2* knock-down (KD) in the dorsomedial prefrontal cortex (dmPFC) does not affect foot shock sensitivity, basal anxiety-like behavior, or associative learning. *Prdm2* KD does not affect shock sensitivity (**A**), locomotor activity (**B**), novel object recognition (**C**) and basal anxiety (**D**).

To assess whether *Prdm2* KD affects fear memory processes, rats received bilateral microinfusions of an AAV that expressed a shRNA targeting *Prdm2* or a scrambled shRNA in the dmPFC (Fig. 1A). *Prdm2* KD resulted in about 50% decreased expression of *Prdm2* in the dmPFC (Fig. 1C, D; One way ANOVA: *F*_(1,17)_ < 0.001, scrambled: *n* = 9 and *Prdm2* KD *n* = 10). Rats were tested for fear memory acquisition, expression, and extinction 1 month after viral infusion (Fig. 1E). We found that *Prdm2* KD in the dmPFC did not affect fear memory acquisition (Fig. 1F), but significantly increased the expression of fear memory 24 h after the conditioning session, as measured by an enhanced tone-evoked freezing response (Fig. 2G, H; One way ANOVA: *F*_(1,35)_ = 11.55; *p* = 0.002; scrambled: *n* = 17 and *Prdm2* KD *n* = 20).

Next, memory extinction was assessed by re-exposing the animals to the expression test, 48 h and 72 h after the conditioning session. Two-way repeated measures ANOVA showed a significant effect of time (*F*_(2,70)_ = 64.2; *P* < 0.001), indicating robust extinction, and a significant effect of group on the cue-evoked freezing response (Fig. 1I; *F*_(1,70)_ = 9.64; *p* = 0.003). However, the slopes of the extinction functions were parallel, reflected in a non-significant time × group interaction (*F*_(2,70)_ = 1.58; *p* = 0.21). Thus, the rate of extinction was not changed by *Prdm2* KD. Although the rate of extinction was not affected, increased fear expression was also observed after 3 days of fear extinction sessions in rats with dmPFC *Prdm2* KD compared to scrambled controls (*F*_(1,35)_ = 4.10; *P* = 0.05). This suggests a persistent effect of *Prdm2* KD on fear memory expression. We then tested the expression of fear memory at a later time point, 1 week after acquisition, to assess long-term effect of *Prdm2* KD. We found that *Prdm2* KD significantly increased the percentage of time spent freezing also at this time point (Fig. 1J; one-way ANOVA: *F*_(1,22)_ = 5.11; *p* < 0.05; scrambled: *n* = 12 and *Prdm2* KD *n* = 12), demonstrating an enduring effect of *Prdm2* KD. Collectively, these data establish an upregulation of conditioned fear responses following *Prdm2* KD, with a time course consistent with an epigenetic reprogramming of the transcriptome.

### *Prdm2* KD increases the expression of cued conditioned fear through effects on memory consolidation

*Prdm2* KD specifically increased fear expression without influencing acquisition of fear (Fig. 1), indicating that PRDM2 does not affect the associative learning processes that link the unconditioned stimulus (i.e., foot shock) with the conditioned stimulus (i.e., tone). *Prdm2* KD may enhance fear expression by modulating processes involved in memory consolidation, or memory recall. To address this question, the AAV containing shRNA against *Prdm2* was infused into the dmPFC one week after fear conditioning. Given the time necessary for the shRNA to stably reduce *Prdm2* expression, most of the consolidation processes had been formed in the dmPFC by this time [13, 41]. Under these conditions, we observed no differences between *Prdm2* KD rats and scrambled controls (Fig. 1K; scrambled: *n* = 20 and *Prdm2* KD *n* = 18), suggesting that *Prdm2* KD in the dmPFC results in a persistent increase in fear expression via effects on memory consolidation, rather than memory recall.

### Effects of *Prdm2* KD in the dmPFC on conditioned fear expression are behaviorally specific

We observed no effects of *Prdm2* KD on foot shock sensitivity and locomotor activity suggesting a specific effect of *Prdm2* KD on fear expression (Fig. 2A, B; scrambled: *n* = 20 and *Prdm2* KD *n* = 20). We also tested whether *Prdm2* KD affects other types of memory, by performing a novel object recognition task and found no effect of *Prdm2* KD in the recognition index, (Fig. 2C; scrambled: *n* = 12 and *Prdm2* KD *n* = 12). The effect of *Prdm2* KD on anxiety-like behavior was also tested. We observed a trend for a decreased percentage open arm time in *Prdm2* KD rats. However, this was not significant, suggesting that *Prdm2* does not robustly affect innate anxiety-like behaviors (Fig. 2D; scrambled: *n* = 20 and *Prdm2* KD *n* = 20).

### *Prdm2* KD increases fear expression through dmPFC -> BLA projections

**Fig. 3:**
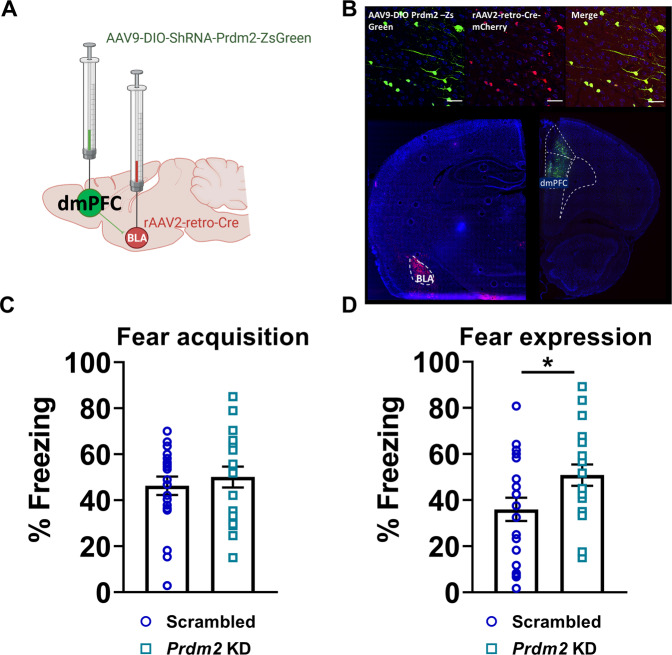
*Prdm2* knock-down (KD) in the dmPFC-BLA projecting neurons increases fear expression. **A** Schematic representing the dual viral approach. **B** Tile scans showing the viral spread in the dmPFC and BLA and representative images showing dmPFC neurons infected by AAV9-DIO-shRNA-PRDM2-ZS green (green), dmPFC neurons infected by rAAV2 retro Cre-mCherry (red) and dmPFC neurons showing co-infection of AAV9-DIO-shRNA-PRDM2-ZS green and rAAV2 retro Cre-mCherry (yellow; scale bar, 50 µm). **C** KD of *Prdm2* in neurons projecting from the dmPFC to the BLA did not affect fear acquisition measured as % freezing ± SEM. **D** *Prdm2* KD in dmPFC-BLA was sufficient to increase fear expression 24 h after conditioning (*N* = 19–20). *p < 0.05. BLA basolateral amygdala, dmPFC dorsomedial prefrontal cortex.

dmPFC -BLA projections are involved in mediating fear expression [12, 13]. We, therefore, hypothesized that *Prdm2* KD may increase fear expression by modulating the activity of dmPFC-BLA projecting neurons. To address this hypothesis, we first investigated whether a selective KD of *Prdm2* in dmPFC-BLA projecting neurons was sufficient to increase fear expression. We used a dual vector approach, in which a retrogradely transported-AAV encoding Cre was injected into the BLA, and an AAV encoding Cre-dependent *Prdm2* microRNA was infused into the dmPFC (Fig. 3A, B) [42]. Similar to what we observed with a *Prdm2* KD in dmPFC that was not projection-specific, *Prdm2* KD in dmPFC-BLA projecting neurons did not affect the acquisition of conditioned fear (Fig. 3C) but significantly increased the expression of fear memory 24 h after acquisition (Fig. 3D; one-way ANOVA: *F*_(1,37)_ = 4.56; *p* < 0.05; scrambled: *n* = 20 and *Prdm2* KD *n* = 19). No differences were seen in locomotor activity or anxiety-like behavior in *Prdm2* KD dmPFC-BLA groups compared to scrambled controls (Supplementary Fig. 4A. B, respectively). A trend for a reduced percentage time spent in the open arm was observed in the dmPFC-BLA *Prdm2* KD group, similar to that observed in the dmPFC *Prdm2* KD group.

### *Prdm2* KD regulates the expression of genes involved in synaptic function

**Fig. 4:**
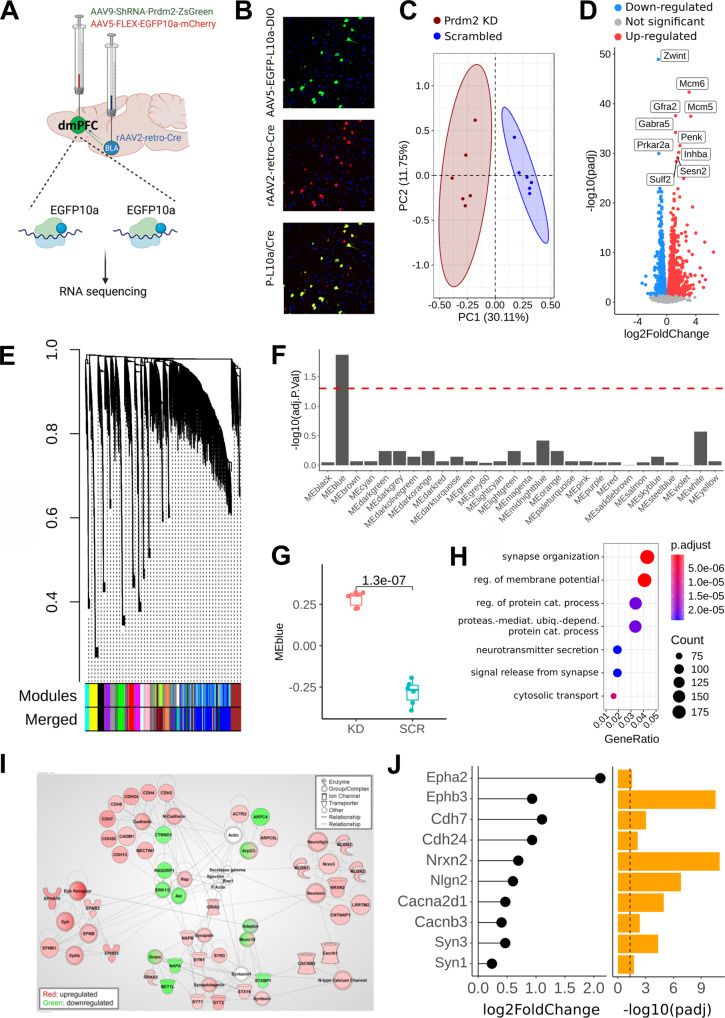
*Prdm2* knock-down (KD) in dmPFC-BLA neurons regulates genes involved in synaptogenesis. *Prdm2* KD in dmPFC-BLA neurons regulates genes involved in synaptogenesis. **A** Schematic representing the triple viral approach used for the vTRAP experiment. **B** Tile scans showing the viral spread in the dmPFC and BLA as well as dmPFC neurons presenting cells infected by AAV5-FLEX-EGFPL10a (green), cells infected by rAAV2 retro Cre-mCherry (red), and cells showing co-infection of AAV5-FLEX-EGFPL10a and rAAV2 retro Cre-mCherry (yellow). **C** Principal component analysis showing separation of *Prdm2* KD samples and scrambled control into distinct clusters. **D** Volcano Plot illustrating the most significantly altered genes following *Prdm2* KD. **E** Hierarchical clustering dendrogram grouping together interconnected, highly co-expressed genes. Colormaps beneath the dendrogram corresponds to modules of co-expressed genes. Top colormap: initial identified modules. Bottom colormap: modules after merging modules with similar expression profiles. **F** Differential expression analysis for each co-expression module, comparing *Prdm2* KD with scrambled control. Red horizontal dashed line denotes a significance level of FDR-corrected *p* value of 0.05. **G** Boxplot comparing the gene expression profile of module “MEblue” between conditions. KD: *Prdm2* KD, SCR: Scramble control. Statistical test: Two-sided unpaired t-test. **H** Gene ontology enrichment for genes in the module “MEblue”. **I** Gene network analysis performed using IPA. *Prdm2* KD increases expression of genes that code for the cadherin, neurexin/neuroligin and ephrin/ephrin receptors family as well as for proteins that belongs to the SNARE complex. **J** Differential expression and significance level for selected synaptogenesis-related genes. Vertical dashed line in the bar plot denotes a significance level of FDR-corrected *p* value of 0.05. BLA basolateral amygdala, dmPFC dorsomedial prefrontal cortex.

To identify the downstream molecular targets through which *Prdm2* KD regulates dmPFC-BLA-increased fear memory consolidation, we sequenced the translatome of dmPFC-BLA neurons using a vTRAP strategy (Fig. 4A, B; scrambled: *n* = 18 rats; pools of 3 dmPFC and *Prdm2* KD *n* = 18 rats; pools of 3 dmPFC) [39].

We found that *Prdm2* KD was sufficient to alter the translational profile of the dmPFC-BLA neurons (Fig. 4C, D). Using vTRAP-RNAseq, we sequenced 22 091 genes and found that *Prdm2* KD modulated the expression of 3603 of these (Supplementary Table 1: 1877 upregulated and 1726 downregulated). As expected *Prdm2* KD decreased the expression of *Prdm2* (Supplementary Table 1: adj *p* = 3.33E-23) in the dmPFC-BLA neurons. WCNA analysis identified a total of 32 co-expression modules of highly correlated genes, which were further merged into 28 distinct modules (Fig. 4E). Among these 28 modules the largest one named “MEblue” showed significant differential expression between *Prdm2* KD and scrambled control (Fig. 4F, G). This module showed enrichment of biological processes such as synapse organization and regulation of membrane potential (Fig. 4H). We also used IPA to cluster genes based on their function. We found that *Prdm2* KD in the dmPFC-BLA neurons modulated the expression of genes that have been associated with fear conditioning, anxiety, emotional behavior, and memory (Supplementary Table 2). In line with the WCNA analysis, the top significant gene network identified by IPA included gene expression changes associated with synaptogenesis activation (Fig. 4I). This network consisted of genes that belong to the ephrin, neuroligin, and neurexin families, as well as SNARE associated genes (e.g., synaptotagmins, Fig. 4I, J). These gene families are known to contribute to neurotransmission and memory formation [43–45].

### *Prdm2* KD in the dmPFC increases glutamate release in the BLA

**Fig. 5:**
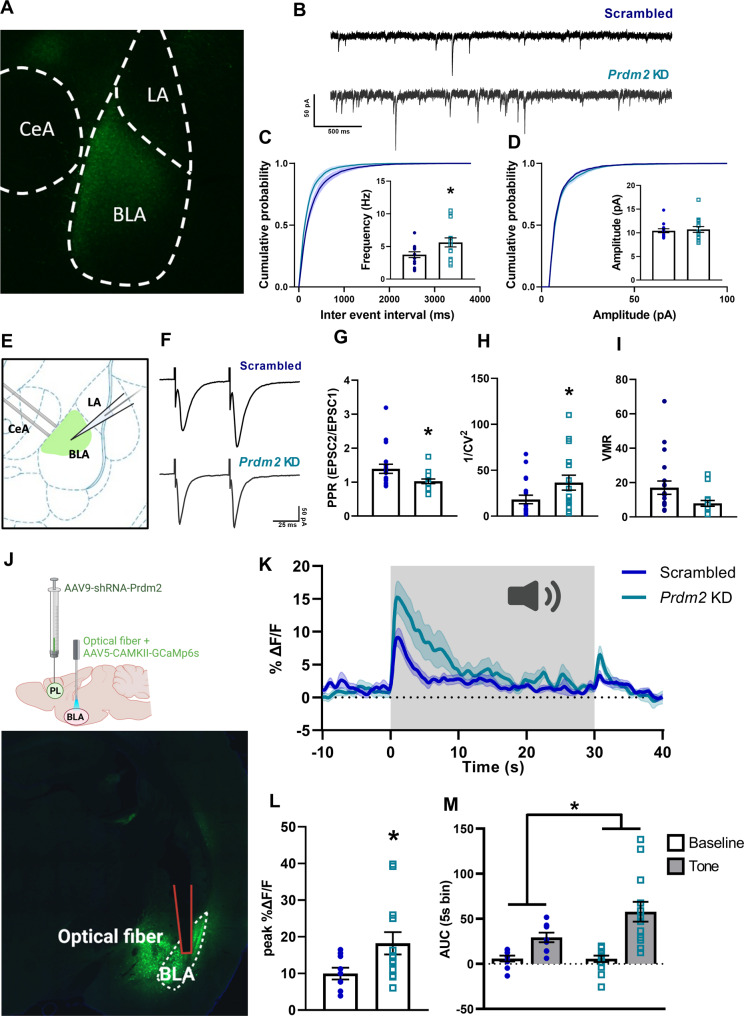
*Prdm2* knock-down (KD) in the dmPFC increases glutamate release probability in the BLA. **A** Representative image showing the viral expression (AAV9.HI.shR.ratPrdm2.CMV.ZsGreen.SV40) in dmPFC terminals targeting the BLA. **B** Representative sEPSCs traces recorded from BLA putative principal neurons (PNs) in Scrambled or *Prdm2* KD rats. Scale bars: 50 pA × 500 ms. Cumulative distributions and bar graphs showing the frequency (**C**) and amplitude (**D**) of sEPSCs recorded from putative BLA PNs in Scrambled or *Prdm2* KD rats. **E** Schematic representation of the recording and stimulating electrodes sites for evoked (e)EPSCs recordings. **F** Representative eEPSCs traces evoked by paired electrical stimulations recorded from putative BLA PNs in Scrambled or *Prdm2* KD rats. Scale bars: 50 pA × 25 ms. **G** Bar graphs showing the mean values of PPR recorded from putative BLA PNs in Scrambled or *Prdm2* KD rats. Bar graphs showing the mean values of 1/*CV*^2^ (**H**) and VMR (**I**) of eEPSCs recorded from Scrambled or *Prdm2* KD rats (*N* = 5–6/group). *p < 0.05. **J** Schematic representing the fiber photometry approach (top) and a representative image (bottom) showing the implanted optical fiber aimed at the BLA and the expression of two viruses, AAV9.HI.shR.ratPrdm2.CMV.ZsGreen.SV40 in dmPFC terminals targeting the BLA as well as AAV9.CaMKII.GCaMP6s.WPRE.SV40 in BLA glutamatergic neurons. **K** Traces showing normalized calcium-dependent GCaMP fluorescent signal (mean ± SEM) in BLA neurons of Scrambled or *Prdm2* KD rats, averaged over the first 2 tones from the fear expression test. Duration of the tone is indicated by the shaded gray box. Bar graphs showing larger peak normalized GCaMP signal in response to tone onset (**L**) and increased Area Under the Curve (AUC) of the normalized GCaMP signal during the 5 s preceding the tone and the first 5 s of the tone presentation (**M**) in *Prdm2* KD rats compared to Scrambled control rats. BLA basolateral amygdala, CeA central amygdala, dmPFC dorsomedial prefrontal cortex, LA lateral amygdala, VMR variance to mean ratio, sEPSCs spontaneous excitatory postsynaptic currents.

The translational reprogramming identified by our RNAseq analysis pointed to the possibility that *Prdm2* KD modulates the consolidation of conditioned fear by modifying synaptic glutamate release at dmPFC -BLA projection targets. To examine this possibility, we carried out slice electrophysiology experiments. We first assessed the effects of dmPFC *Prdm2* KD on BLA basal synaptic properties by measuring sEPSCs, recorded from putative BLA principal neurons in scrambled controls and *Prdm2* KD rats (Fig. 5A). *Prdm2* KD rats showed an increased sEPSCs frequency compared to scrambled controls (Fig. 5B, C; Scrambled: 3.74 ± 0.45 Hz, *n* = 14; *Prmd2* KD: 5.63 ± 0.70 Hz, *n* = 15; *t*(27) = 2.24, *p* < 0.05, unpaired *t* test). In contrast, *Prdm2* KD rats did not show significant differences in the amplitude (Fig. 5D; Scrambled: 10.45 ± 0.41 pA, *n* = 14; *Prmd2* KD: 10.71 ± 0.61 pA, *n* = 15; *t*(27) = 0.35, *p* = 0.73; unpaired *t* test) and kinetics (Rise time: Scrambled: 2.16 ± 0.07 ms, *n* = 14; *Prmd2* KD: 2.15 ± 0.06 ms, *n* = 15; *t*(27) = 0.08 *p* = 0.94, unpaired *t* test; Decay time: Scrambled: 10.93 ± 0.51 ms, *n* = 14; *Prmd2* KD: 10.43 ± 0.31 ms, *n* = 15; *t*(27)=0.85 *p* = 0.40, unpaired t-test; data not shown) of sEPSCs. These data suggest that *Prdm2* KD in the dmPFC increased glutamate release onto BLA principal neurons.

To investigate whether *Prdm2* KD affects release probability, we compared the paired-pulse ratio (PPR) of electrically eEPSCs recorded from putative BLA principal neurons from scrambled controls and *Prdm2* KD rats (Fig. 5E, F). Consistent with an enhanced release probability of the BLA glutamatergic inputs, we observed a lower PPR in *Prdm2* KD rats compared to scrambled controls (Fig. 5G; Scrambled: 1.39 ± 0.13, *n* = 19; *Prdm2* KD: 1.02 ± 0.07, *n* = 16; *p* < 0.05, Mann Whitney test). Furthermore, to provide an independent measure of changes in release probability, we analyzed the alterations induced by *Prdm2* KD on the inverse square of the coefficient of variation (1/*CV*^2^) and the VMR of eEPSCs [46]. We found that *Prdm2* KD rats exhibit a significantly higher value of 1/*CV*^2^ (Fig. 5H; Scrambled: 18.14 ± 4.62, *n* = 19; *Prmd2* KD: 36.46 ± 8.08, *n* = 16; *p* < 0.05, Mann Whitney test), and a nearly significant lower value of VMR (Fig. 5I; Scrambled: 17.04 ± 3.90, *n* = 19; *Prmd2* KD: 7.91 ± 1.69, *n* = 16; *p* = 0.08, Mann Whitney test). Although concurrent changes in the number of functional vesicles releasing sites cannot be excluded, these data appear consistent with the PPR results further suggesting an increased release probability of the glutamatergic inputs to the BLA following dmPFC *Prdm2* KD. Last, we measured AMPA/NMDA ratio to determine whether *Prdm2* KD could also alter the relative functional expression of BLA AMPA and NMDA glutamatergic receptors. *Prdm2* KD failed to alter the AMPA/NMDA ratio (Supplementary Fig. 5; Scrambled: 2.97 ± 0.60, *n* = 12; *Prmd2* KD: 2.76 ± 0.33, *n* = 12; *p* = 0.86, Mann Whitney test), indicating the absence of postsynaptic remodeling in the glutamatergic synapses converging to the BLA in response to *Prdm2* KD.

### *Prdm2* KD in the dmPFC increases BLA neuronal activity in response to a fear-associated tone

Our behavioral and electrophysiology data suggested that *Prdm2* KD may be potentiating the expression of cued conditioned fear by increasing reactivity of BLA pyramidal neurons to fear-associated cues. To test this hypothesis, we used fiber photometry to measure BLA neuronal activity during expression of fear memory (scrambled: *n* = 9 and *Prdm2* KD *n* = 13). An AAV-vector encoding the fluorescent calcium sensor GCaMP6s under the control of the CaMKII promotor was injected unilaterally into the BLA of *Prdm2* KD rats and scrambled controls, followed by implantation of an optical fiber (Fig. 5J). Rats underwent a fear conditioning session and were tested 24 h later for cue-induced expression of conditioned fear, as before, and calcium activity in the BLA was measured during the test session. Confirming our hypothesis, larger changes in GCaMP fluorescence were observed during the first two presentations of the fear-associated tone in *Prdm2* KD rats compared to scrambled controls (Fig. 5K). This effect was observed both when measuring peak normalized GCaMP signal in response to tone onset (Fig. 5L; *t*(20) = 2.37, *p* = 0.03) as well as when calculating the Area Under the Curve (AUC) of the normalized GCaMP signal during the 5 s preceding the tone and the first 5 s of the tone presentation (Fig. 5M; treatment effect: *F*(1,20) = 5.28; *p* = 0.03).

## Discussion

We report a mechanistic role of the epigenetic enzyme PRDM2 in modulating fear memory consolidation through the regulation of the dmPFC-BLA neuronal pathway. We show that enhanced fear expression after *Prdm2* KD may result from an increased expression of genes involved in neurotransmitter release, leading to a heightened activity of the glutamatergic dmPFC-BLA projecting neurons during fear memory recall. Collectively, our findings provide a novel molecular mechanism through which the dmPFC-BLA projection may promote excessive and enduring fear response.

Substantial evidence supports a role of prefrontal cortex-amygdala circuits in the regulation of fear and anxiety [12–14]. Functional imaging studies in humans show an increased functional connectivity between the dmPFC/anterior cingulate cortex (ACC) and the amygdala during threat processing in healthy individuals [47]. In individuals with generalized or social anxiety disorders, patients with the most severe symptoms also show the highest dmPFC/ACC-amygdala connectivity, suggesting that this circuit is not only involved in adaptive but also in maladaptive responses to stress [48]. Consistent with a role of the dmPFC/ACC-amygdala pathway in adaptative stress responses, animal research shows that fear expression critically relies on activation of the dmPFC-BLA pathway [11]. Cued conditioned fear strengthens the dmPFC excitatory synapses in the BLA [12], and optogenetic silencing of the dmPFC terminals in the BLA decreases both fear expression and active avoidance [13, 49].

Our findings are consistent with these observations and provide a potential molecular mechanism for how maladaptive fear responses may arise. We show that *Prdm2* KD in the dmPFC-BLA pathway is sufficient to potentiate expression of conditioned fear. We also found that *Prdm2* KD induces profound changes in the translational profile of dmPFC-BLA neurons and promotes increased glutamatergic neurotransmission in the BLA. Specifically, *Prdm2* KD modified the translational profile of 100 genes involved in synaptogenesis, suggesting that PRDM2 plays an important role in regulating synaptic functions. *Prdm2* KD increased expression of genes coding for the SNARE complex (synaptotagmins and syntaxins) and N-type calcium channels. Additionally, *Prdm2* KD increased the expression of genes belonging to cell adhesion protein families (i.e., neurexins; neuroligins; ephrins) which are known to play an important role in synaptic transmission and synaptic plasticity [44, 45, 50]. It is important to note that in the vTRAP approach, only mRNAs that are associated with ribosomes (i.e., that are in the process of being translated), are sequenced. Translating mRNAs are thus more closely correlated with protein levels, which makes changes in translating mRNA following *Prdm2* KD more likely to functionally impact neuronal activity and consequently fear memory processes. Our translatomic findings strongly indicate that *Prdm2* KD facilitates neurotransmitter release by increasing the expression of genes that control synaptic vesicle fusion. This hypothesis is supported by our patch-clamp recordings which indicate an enhanced neurotransmitter release probability in glutamatergic inputs to the BLA in *Prdm2* KD rats compared to scrambled controls. Additionally, *Prdm2* KD rats showed an increased neuronal activity in the BLA during fear expression, suggesting that *Prdm2* KD may enhance fear expression through increased synaptic efficacy of the dmPFC-BLA projecting neurons.

Our data also indicate that *Prdm2* KD potentiates fear expression by increasing fear memory consolidation, a process through which newly acquired memories stabilize to form long-term memory [51]. The strength of a memory consolidation can influence individual variation in fear responses. For instance, “over-consolidation” of a trauma-associated memory may induce a stronger response and render an individual more prone to develop trauma-related pathological anxiety [52, 53]. In line with a role of *Prdm2* in fear memory consolidation, several genes that are modulated by *Prdm2* KD, including brain-derived neurotrophic factor and the Cyclic AMP-Responsive Element-Binding Protein 1 gene were found to be associated with fear memory consolidation [54–57]. Additionally, a recent study from Chen et al. [58] showed that fear memory consolidation is associated with increased expression of genes that code for proteins of the SNARE complex, and for several neuroligins and neurexins. In what could be likened with a priming mechanism, increased expression of these genes prior to fear exposure may contribute to subsequent over-consolidation of fear memories. Because prolonged exposure of the brain to alcohol down-regulates *Prdm2* expression in the dmPFC [23], our findings also provide a candidate mechanism for increased vulnerability to pathological over-consolidation of fear memories in people with alcohol use disorders, consistent with the high co-morbidity of excessive alcohol use and PTSD [59].

In conclusion, we propose a novel mechanism for increased fear memory consolidation, wherein decreased *Prdm2* expression in the dmPFC-BLA projecting neurons results in translatomic changes that promote increased synaptic efficacy in the BLA and increased dmPFC-BLA neuronal responses to stress-associated cues. This study also provides the first set of evidence for a role of PRDM2 in stress-related disorders. Finally, given our prior findings that alcohol dependence down-regulates dmPFC *PRDM2* expression [23], we provide a candidate mechanism for the extensive co-morbidity of alcohol use and anxiety disorders.

## Supplementary information

Supplementary figures

Supplementary figure and table legends

Supplementary methods

## References

[1] Ohman A (1986). Face the beast and fear the face: animal and social fears as prototypes for evolutionary analyses of emotion. Psychophysiology.

[2] Epstein S. The nature of anxiety with emphasis upon its relationship to expectancy. Vol. 2: New ‘Vbrk: Academic Press; 1972.

[3] Craske MG, Stein MB, Eley TC, Milad MR, Holmes A, Rapee RM (2017). Anxiety disorders. Nat Rev Dis Prim.

[4] Lissek S, van Meurs B (2015). Learning models of PTSD: theoretical accounts and psychobiological evidence. Int J Psychophysiol.

[5] LeDoux JE (2000). Emotion circuits in the brain. Annu Rev Neurosci.

[6] Ciocchi S, Herry C, Grenier F, Wolff SB, Letzkus JJ, Vlachos I (2010). Encoding of conditioned fear in central amygdala inhibitory circuits. Nature.

[7] Davis M, Whalen PJ (2001). The amygdala: vigilance and emotion. Mol Psychiatry.

[8] Burgos-Robles A, Kimchi EY, Izadmehr EM, Porzenheim MJ, Ramos-Guasp WA, Nieh EH (2017). Amygdala inputs to prefrontal cortex guide behavior amid conflicting cues of reward and punishment. Nat Neurosci.

[9] Damasio H, Grabowski T, Frank R, Galaburda AM, Damasio AR (1994). The return of Phineas Gage: clues about the brain from the skull of a famous patient. Science.

[10] Gilmartin MR, Balderston NL, Helmstetter FJ (2014). Prefrontal cortical regulation of fear learning. Trends Neurosci.

[11] Sierra-Mercado D, Padilla-Coreano N, Quirk GJ (2011). Dissociable roles of prelimbic and infralimbic cortices, ventral hippocampus, and basolateral amygdala in the expression and extinction of conditioned fear. Neuropsychopharmacology.

[12] Arruda-Carvalho M, Clem RL (2014). Pathway-selective adjustment of prefrontal-amygdala transmission during fear encoding. J Neurosci.

[13] Do-Monte FH, Quinones-Laracuente K, Quirk GJ (2015). A temporal shift in the circuits mediating retrieval of fear memory. Nature.

[14] Kim MJ, Loucks RA, Palmer AL, Brown AC, Solomon KM, Marchante AN (2011). The structural and functional connectivity of the amygdala: from normal emotion to pathological anxiety. Behav Brain Res.

[15] Descalzi G, Li XY, Chen T, Mercaldo V, Koga K, Zhuo M (2012). Rapid synaptic potentiation within the anterior cingulate cortex mediates trace fear learning. Mol Brain.

[16] Zovkic IB, Sweatt JD (2013). Epigenetic mechanisms in learned fear: implications for PTSD. Neuropsychopharmacology.

[17] Monsey MS, Ota KT, Akingbade IF, Hong ES, Schafe GE (2011). Epigenetic alterations are critical for fear memory consolidation and synaptic plasticity in the lateral amygdala. PLoS One.

[18] Jhang J, Lee H, Kang MS, Lee HS, Park H, Han JH (2018). Anterior cingulate cortex and its input to the basolateral amygdala control innate fear response. Nat Commun.

[19] Weaver IC, Cervoni N, Champagne FA, D’Alessio AC, Sharma S, Seckl JR (2004). Epigenetic programming by maternal behavior. Nat Neurosci.

[20] Murgatroyd C, Spengler D (2011). Epigenetics of early child development. Front Psychiatry.

[21] Borrelli E, Nestler EJ, Allis CD, Sassone-Corsi P (2008). Decoding the epigenetic language of neuronal plasticity. Neuron.

[22] Sorrentino A, Rienzo M, Ciccodicola A, Casamassimi A, Abbondanza C (2018). Human PRDM2: structure, function and pathophysiology. Biochim Biophys Acta Gene Regul Mech..

[23] Barbier E, Johnstone AL, Khomtchouk BB, Tapocik JD, Pitcairn C, Rehman F (2017). Dependence-induced increase of alcohol self-administration and compulsive drinking mediated by the histone methyltransferase PRDM2. Mol Psychiatry.

[24] Scarlata MJ, Lee SH, Lee D, Kandigian SE, Hiller AJ, Dishart JG (2019). Chemogenetic stimulation of the infralimbic cortex reverses alcohol-induced fear memory overgeneralization. Sci Rep.

[25] Burns L, Teesson M (2002). Alcohol use disorders comorbid with anxiety, depression and drug use disorders. Findings from the Australian National Survey of Mental Health and Well Being. Drug Alcohol Depend.

[26] Hasin DS, Stinson FS, Ogburn E, Grant BF (2007). Prevalence, correlates, disability, and comorbidity of DSM-IV alcohol abuse and dependence in the United States: results from the National Epidemiologic Survey on Alcohol and Related Conditions. Arch Gen Psychiatry.

[27] Heilig M, Barbier E, Johnstone AL, Tapocik J, Meinhardt MW, Pfarr S (2017). Reprogramming of mPFC transcriptome and function in alcohol dependence. Genes Brain Behav.

[28] Park J, Wood J, Bondi C, Del Arco A, Moghaddam B (2016). Anxiety evokes hypofrontality and disrupts rule-relevant encoding by dorsomedial prefrontal cortex neurons. J Neurosci.

[29] Rozeske RR, Jercog D, Karalis N, Chaudun F, Khoder S, Girard D (2018). Prefrontal-periaqueductal gray-projecting neurons mediate context fear discrimination. Neuron.

[30] Sewards TV, Sewards MA (2002). Fear and power-dominance drive motivation: neural representations and pathways mediating sensory and mnemonic inputs, and outputs to premotor structures. Neurosci Biobehav Rev.

[31] Antunes M, Biala G (2012). The novel object recognition memory: neurobiology, test procedure, and its modifications. Cogn Process.

[32] Pellow S, Chopin P, File SE, Briley M (1985). Validation of open:closed arm entries in an elevated plus-maze as a measure of anxiety in the rat. J Neurosci Methods.

[33] Domi E, Barbier E, Augier E, Augier G, Gehlert D, Barchiesi R (2018). Preclinical evaluation of the kappa-opioid receptor antagonist CERC-501 as a candidate therapeutic for alcohol use disorders. Neuropsychopharmacology.

[34] Paxinos GWC. The rat brain in stereotaxic coordinates, vol. 6th ed. Waltham, MA: Academic Press; 2007.

[35] Lerner TN, Shilyansky C, Davidson TJ, Evans KE, Beier KT, Zalocusky KA (2015). Intact-brain analyses reveal distinct information carried by SNc dopamine subcircuits. Cell.

[36] Barker DJ, Miranda-Barrientos J, Zhang S, Root DH, Wang HL, Liu B (2017). Lateral preoptic control of the lateral habenula through convergent glutamate and GABA transmission. Cell Rep.

[37] Braunscheidel KM, Okas MP, Hoffman M, Mulholland PJ, Floresco SB, Woodward JJ (2019). The abused inhalant toluene impairs medial prefrontal cortex activity and risk/reward decision-making during a probabilistic discounting task. J Neurosci.

[38] Heiman M, Kulicke R, Fenster RJ, Greengard P, Heintz N (2014). Cell type-specific mRNA purification by translating ribosome affinity purification (TRAP). Nat Protoc.

[39] Nectow AR, Moya MV, Ekstrand MI, Mousa A, McGuire KL, Sferrazza CE (2017). Rapid molecular profiling of defined cell types using viral TRAP. Cell Rep.

[40] Rubio FJ, Liu QR, Li X, Cruz FC, Leao RM, Warren BL (2015). Context-induced reinstatement of methamphetamine seeking is associated with unique molecular alterations in Fos-expressing dorsolateral striatum neurons. J Neurosci.

[41] Johansen JP, Cain CK, Ostroff LE, LeDoux JE (2011). Molecular mechanisms of fear learning and memory. Cell.

[42] Barbier E, Barchiesi R, Domi A, Chanthongdee K, Domi E, Augier G (2021). Downregulation of synaptotagmin 1 in the prelimbic cortex drives alcohol-associated behaviors in rats. Biol Psychiatry.

[43] Chen F, Chen H, Chen Y, Wei W, Sun Y, Zhang L (2021). Dysfunction of the SNARE complex in neurological and psychiatric disorders. Pharmacol Res.

[44] Sudhof TC (2008). Neuroligins and neurexins link synaptic function to cognitive disease. Nature.

[45] Dines M, Lamprecht R. The role of ephs and ephrins in memory formation. Int J Neuropsychopharmacol. 2016;19:1–14. 10.1093/ijnp/pyv106 PMC4851260 26371183

[46] van Huijstee AN, Kessels HW (2020). Variance analysis as a tool to predict the mechanism underlying synaptic plasticity. J Neurosci Methods.

[47] Robinson OJ, Charney DR, Overstreet C, Vytal K, Grillon C (2012). The adaptive threat bias in anxiety: amygdala-dorsomedial prefrontal cortex coupling and aversive amplification. Neuroimage.

[48] Robinson OJ, Krimsky M, Lieberman L, Allen P, Vytal K, Grillon C (2014). Towards a mechanistic understanding of pathological anxiety: the dorsal medial prefrontal-amygdala ‘aversive amplification’ circuit in unmedicated generalized and social anxiety disorders. Lancet Psychiatry.

[49] Choi DC, Gourley SL, Ressler KJ (2012). Prelimbic BDNF and TrkB signaling regulates consolidation of both appetitive and aversive emotional learning. Transl Psychiatry.

[50] Missler M, Zhang W, Rohlmann A, Kattenstroth G, Hammer RE, Gottmann K (2003). Alpha-neurexins couple Ca2+ channels to synaptic vesicle exocytosis. Nature.

[51] Alberini CM, Milekic MH, Tronel S (2006). Mechanisms of memory stabilization and de-stabilization. Cell Mol Life Sci.

[52] Morrison FG, Ressler KJ (2014). From the neurobiology of extinction to improved clinical treatments. Depress Anxiety.

[53] Kindt M (2014). A behavioural neuroscience perspective on the aetiology and treatment of anxiety disorders. Behav Res Ther.

[54] Choi DC, Maguschak KA, Ye K, Jang SW, Myers KM, Ressler KJ (2010). Prelimbic cortical BDNF is required for memory of learned fear but not extinction or innate fear. Proc Natl Acad Sci USA.

[55] Rao-Ruiz P, Couey JJ, Marcelo IM, Bouwkamp CG, Slump DE, Matos MR (2019). Engram-specific transcriptome profiling of contextual memory consolidation. Nat Commun.

[56] Lubin FD, Roth TL, Sweatt JD (2008). Epigenetic regulation of BDNF gene transcription in the consolidation of fear memory. J Neurosci.

[57] Matos MR, Visser E, Kramvis I, van der Loo RJ, Gebuis T, Zalm R (2019). Memory strength gates the involvement of a CREB-dependent cortical fear engram in remote memory. Nat Commun.

[58] Chen MB, Jiang X, Quake SR, Sudhof TC (2020). Persistent transcriptional programmes are associated with remote memory. Nature.

[59] Kessler RC, Nelson CB, McGonagle KA, Edlund MJ, Frank RG, Leaf PJ (1996). The epidemiology of co-occurring addictive and mental disorders: implications for prevention and service utilization. Am J Orthopsychiatry.

