# Correction: An epigenetic mechanism for over-consolidation of fear memories

**DOI:** 10.1038/s41380-022-01827-w

**Published:** 2022-11-01

**Authors:** Riccardo Barchiesi, Kanat Chanthongdee, Michele Petrella, Li Xu, Simon Söderholm, Esi Domi, Gaelle Augier, Andrea Coppola, Joost Wiskerke, Ilona Szczot, Ana Domi, Louise Adermark, Eric Augier, Claudio Cantù, Markus Heilig, Estelle Barbier

**Affiliations:** 1 Center for Social and Affective Neuroscience, Department of Biomedical and Clinical Sciences, Linköping University, S-581 85 Linköping, Sweden; 2 Department of Physiology, Faculty of Medicine Siriraj Hospital, Mahidol University, Bangkok, Thailand; 3 Wallenberg Centre for Molecular Medicine, Linköping University, Linköping, Sweden; 4 Department of Biomedical and Clinical Sciences, Division of Molecular Medicine and Virology; Faculty of Medicine and Health Sciences, Linköping University, Linköping, Sweden; 5 Addiction Biology Unit, Department of Psychiatry and Neurochemistry, Institute of Neuroscience and Physiology, The Sahlgrenska Academy, University of Gothenburg, Gothenburg, Sweden

**Keywords:** Neuroscience, Molecular biology

Correction to: *Molecular Psychiatry* 10.1038/s41380-022-01758-6, published online 21 September 2022

In the original version of this article, the given and family names of Riccardo Barchiesi, Kanat Chanthongdee, Michele Petrella, Li Xu, Simon Söderholm, Esi Domi, Gaelle Augier, Andrea Coppola, Joost Wiskerke, Ilona Szczot, Ana Domi, Louise Adermark, Eric Augier, Claudio Cantù, Markus Heilig, Estelle Barbier were incorrectly structured. The names were displayed correctly in all versions at the time of publication.

The original article has been corrected.

