# Supplementary material for: An epigenetic mechanism for over-consolidation of fear memories
Source: Mol Psychiatry. 2022 Sep 21;27(12):4893–904. doi: 10.1038/s41380-022-01758-6 (PMC9763112; doi:10.1038/s41380-022-01758-6)
Supplement: Supplementary file 2 — Supplementary figure and table legends [file 41380_2022_1758_MOESM2_ESM.docx]

**Supplementary legends:**

**Supplementary Fig. 1. Overview of experimental design and timelines.** Eleven batches of rats were used in this study (N=268). A) Detailed description of the fear conditioning paradigm. B) Timeline for each experiment. KD: knock-down. Figure was created with BioRender.com.

**Supplementary Fig. 2. Effects of *Prdm2* KD on conditioned fear are specific for cue-induced expression of fear memories.** No differences were observed in *Prdm2* KD rats compared to scrambled controls on (A) plasma corticosterone levels or (B) context generalization. (C) The increased cue-induced expression of fear following Prdm2 KD was replicated in a separate batch of animals (One way ANOVA: F(1,37)=16.6; P<0.001) ; experiment 3) and (D) the efficiency of the viral vector-mediated KD was also assessed by qPCR in this batch. dmPFC: dorsomedial cortex. ***p<0.001

**Supplementary Fig 3. Custom-made objects used for the novel object recognition.** Objects were made interactive (climbable) in order to increase exploration time and memory acquisition, as novel object recognition is normally used to study short term memory.

**Supplementary Fig. 4. Projection-specific KD of *Prdm2* does not affect anxiety-like behavior or locomotor activity.** KD of *Prdm2* in PL-BLA neurons does not affect anxiety-like behavior as measured in (A) the elevated plus maze (EPM) or (B) locomotor activity, although a trend was observed for *Prdm2* KD to decrease percentage time spent in the open arm (OA). dmPFC: dorsomedial cortex; BLA: basolateral amygdala.

**Supplementary Figure 5: *Prdm2* KD in the dmPFC does not affect AMPA/NMDA ratio in the BLA.**

(A) Representative traces of EPSCs evoked by electrical stimulation recorded at -70 and +40 mV to measure the ratio between the amplitude of AMPA and NMDA receptor components of glutamatergic transmission recorded from putative BLA PNs in Scrambled or *Prdm2* KD rats. Scale bars: 200 pA x 50 ms. (B) Bar graphs showing the mean values of AMPA/NMDA ratio recorded from putative BLA PNs in Scrambled or *Prdm2* KD rats

**Supplementary Fig. 6.** (A) Scree plot detailing the percentage explained variance by each principal component in the analysis of the RNAseq data. (B) Graph detailing the 20 most significantly up- and downregulated genes following *Prdm2* KD in the PL-BLA projecting neurons. (B) graph showing RNA concentration after enrichment of EGFPL10a bound RNA using anti GFP. Control samples that do not contain EGFPL10a show negligeable amount of mRNA after RNA purification compared to EGFPL10a samples.

**Supplementary Fig. 7. *Prdm2* KD does not affect anxiety-like behavior after foot-shock exposure.** (A) anxiety-like behavior measured as % time spent in the open arm 15 min after foot-shock exposure in scrambled control and *Prdm2* KD rats. (B) anxiety-like behavior measured as % time spent in the open arm 15 min after foot-shock exposure in scrambled control and *Prdm2* KD rats.

**Supplementary Table 1**: List of genes that are differently expressed in Prdm2 KD compared to scrambled control rats.

**Supplementary Table 2**: List of diseases and functions associated with differently expressed genes (Prdm2 KD vs. scrambled control rats, Ingenuity Pathway Analysis®; Qiagen)
