# Supplementary material for: An epigenetic mechanism for over-consolidation of fear memories
Source: Mol Psychiatry. 2022 Sep 21;27(12):4893–904. doi: 10.1038/s41380-022-01758-6 (PMC9763112; doi:10.1038/s41380-022-01758-6)
Supplement: Supplementary file 3 — Supplementary methods [file 41380_2022_1758_MOESM3_ESM.docx]

**Supplementary methodologies**

**BEHAVIORAL TESTING**

Nine batches of rats were used in this study (N=268). Animal grouping was assigned randomly. In experiment 1 (n=17 scrambled and 20 *Prdm2* KD), rats were tested for acquisition, expression (after 24h) and extinction of fear memory. In experiment 2 (scrambled: n=12 and *Prdm2* KD n=12), rats were tested for expression of fear memory 1 week after conditioning as well as for context generalization and foot shock sensitivity. In experiment 3 (n=17 scrambled and 20 *Prdm2* KD), we replicated the effect of *Prdm2* KD increased fear expression 24h following cued fear conditioning. Prior to undergoing fear conditioning, rats were tested for anxiety in the elevated plus maze (EPM) and locomotor activity. Plasma corticosterone levels were measured at baseline, after conditioning and after testing the expression of fear memory. 1 week after the fear expression test, rats were euthanized, and the PL was collected for gene expression analysis. In experiment 4 (scrambled: n=20 and *Prdm2* KD n=18), rats were conditioned to the fear stimulus 1 week prior to the viral-mediated KD of *Prdm2* and tested for fear expression one month after the surgery. In experiment 5 (scrambled: n=12 and *Prdm2* KD n=12), rats were tested for novel object recognition. In experiment 6 (scrambled: n=20 and *Prdm2* KD n=19), the effects of *Prdm2* KD in neurons specifically projecting to the BLA was investigated on the expression of fear memory 24h after conditioning. In experiment 7 (scrambled: n=18 rats; pools of 3 PL and *Prdm2* KD n=18 rats; pools of 3 PL), we used viral translating ribosomal affinity purification (vTRAP) to analyze gene expression following Prdm2 knock-down specifically in the neurons projecting from the PL to the BLA. In experiments 8 (scrambled: n=14 cells from 5 rats and *Prdm2* KD n=15 cells from 6 rats) we used ex vivo electrophysiology to investigate changes in glutamate release to BLA neurons following *Prdm2* KD. Finally, in experiment 9 (scrambled: n=9 and *Prdm2* KD n=13) we used in vivo fiber photometry to further investigate the consequences of *Prdm2* KD in the BLA. 11 scrambled and 7 *Prdm2* KD were excluded due to fiber misplacement or flat line in GCaMP signal. An overview of the experiments conducted in this study is given in Supplementary Fig. 1.

**Dorsomedial prelimbic cortex dissection:**

Dorsomedial prefrontal cortex was collected at the dmPFC level (-3.7 to -2.7 from bregma) using a 2mm diameter punch.

**Cued fear conditioning contexts**

Context A had a white house-light, a patterned wall and bedding was sprayed with a potassium peroxymonosulfate compound (OXONE®; Merck & Co., Inc, Kenilworth, NJ, USA) immediately before the session start. Context B had a green and a red cue light, and bedding was sprayed with 70% ethanol immediately before session start.

**qPCR**

After completion of experiment 2, brains were removed, flash frozen and kept at -80°C. The PL was collected using cryosectioning and a 2 mm micro punch needle. RNA was extracted using Quick-RNA Microprep kits following manufacturer’s manual (Zymo Research, Irvine, CA, USA). RNA was converted to cDNA using TaqMan cDNA synthesis Kit and qPCR was performed using TaqMan Fast Advanced Master Mix, according to manufacturer’s instructions. cDNA was analyzed on a 7900 PCR with SDS 2.4.2 software (Thermo Fisher Scientific). To measure *Prdm2*, we used inventoried TaqMan Gene expression assay probes (Rn01516793_m1; Life Technologies, Carlsbad, CA). Gene expression was measured with respect to *Gapdh* (Rn01775763_g1) using 2^-ΔΔCt^ analysis ^57^.

**Plasma corticosterone analysis**

Blood samples were collected from tail veins at baseline, 10 min after conditioning and 10 min after expression test into heparin coated tubes and centrifuged for 5 min at 2000 x g to separate plasma. Plasma was transferred into new tubes and stored at -80°C until further analysis. Corticosterone was extracted by adding 5 parts of ethyl acetate (Thermo Fisher Scientific Inc. Waltham, MA, US) to each plasma sample. The layer with organic solvent was first transferred to a water-prefilled tube and then to second tube. This procedure was repeated two times before samples were dried in a vacuum concentrator. Samples were re-dissolved in Assay buffer from the DetectX Corticosterone Enzyme Immunoassay Kit (Arbor Assays, Ann Arbor, MI, USA) and the manufacturer’s protocol was followed.
